# Supplementary material for: A nomogram based on biparametric magnetic resonance imaging for detection of clinically significant prostate cancer in biopsy-naïve patients
Source: Cancer Imaging. 2023 Sep 4;23:82. doi: 10.1186/s40644-023-00606-2 (PMC10478308; doi:10.1186/s40644-023-00606-2)
Supplement: Supplementary file 1 — Supplementary Material 1 [file 40644_2023_606_MOESM1_ESM.docx]

***Title Page***

***Title*: A Nomogram based on Biparametric Magnetic Resonance Imaging for Detection of Clinically Significant Prostate Cancer in Biopsy-Naïve Patients**

***Authors***:

Beibei Hu, Department of Medical Imaging, Jiangsu Vocational College of Medicine, Yancheng, China

E-mail: submitfor_sci@126.com

Huili Zhang, Department of Medical Imaging, Jiangsu Vocational College of Medicine, Yancheng, China

E-mail: [ee21002026@163.com](mailto:ee21002026@163.com)

Yueyue Zhang, Department of Radiology, Second Affiliated Hospital of Soochow University, Soochow, China

E-mail: [a1190108@126.com](mailto:a1190108@126.com)

Yongming Jin, Affiliated Yancheng Hospital, School of Medicine, Southeast University; Yancheng Third People's Hospital, Yancheng, China

E-mail: [15189210300@163.com](mailto:15189210300@163.com)

***Correspondence****:*

Beibei Hu, Department of Medical Imaging, Jiangsu Vocational College of Medicine, Yancheng, China

E-mail: <submitfor_sci@126.com>

Yongming Jin, Affiliated Yancheng Hospital, School of Medicine, Southeast University; Yancheng Third People's Hospital, Yancheng, China

E-mail: [15189210300@163.com](mailto:15189210300@163.com)

**A Nomogram based on Biparametric Magnetic Resonance Imaging for Detection of Clinically Significant Prostate Cancer in Biopsy-Naïve Patients**

**Purpose:** This study aimed to develop and validate a model based on biparametric magnetic resonance imaging (bpMRI) for the detection of clinically significant prostate cancer (csPCa) in biopsy-naïve patients.

**Method**: This retrospective study included 324 patients who underwent bpMRI and MRI targeted fusion biopsy (MRGB) and/or systematic biopsy, of them 217 were randomly assigned to the training group and 107 were assigned to the validation group. We assessed the diagnostic performance of three bpMRI-based scorings in terms of sensitivity and specificity. Subsequently, 3 models (Model 1, Model 2, and Model 3) combining bpMRI scorings with clinical variables were constructed and compared with each other using the area under the receiver operating characteristic (ROC) curves (AUC). The statistical significance of differences among these models was evaluated using DeLong’s test.

**Results**: In the training group, 68 of 217 patients had pathologically proven csPCa. The sensitivity and specificity for Scoring 1 were 64.7% (95% CI 52.2%-75.9%) and 80.5% (95% CI 73.3%-86.6%); for Scoring 2 were 86.8% (95% CI 76.4%-93.8%) and 73.2% (95% CI 65.3%-80.1%); and for Scoring 3 were 61.8% (95% CI 49.2%-73.3%) and 80.5% (95% CI 73.3%-86.6%), respectively. Multivariable regression analysis revealed that scorings based on bpMRI, age, and prostate-specific antigen density (PSAD) were independent predictors of csPCa. The AUCs for the 3 models were 0.88 (95% CI 0.83-0.93), 0.90 (95% CI 0.85-0.94), and 0.88 (95% CI 0.83-0.93), respectively. Model 2 showed significantly higher performance than Model 1 (P=0.03) and Model 3 (P<0.01).

**Conclusion**: All three scorings had favorite diagnostic accuracy. While in conjunction with age and PSAD the prediction power was significantly improved, and the Model 2 that based on Scoring 2 yielded the highest performance.

***Keywords***: bpMRI; prostate cancer; diagnosis; model; nomogram

**Introduction**

Prostate cancer (PCa) is one of the most common malignant cancer in man. It is estimated that approximately 1.3 million new cases worldwide every year, and currently about 10 million people are living with PCa (1,2). MRI plays an important role in localizing, diagnosis, and staging of PCa (3–5); moreover, previous studies demonstrated that MRGB is superior to conventional standard transrectal ultrasonography (TRUS)–guided biopsy, which could considerably reduce the risk of upgrading Gleason Score (GS) 3+4 lesions as compared to standard biopsy (6). In 2019, the American College of Radiology and the European Society of Urogenital Radiology updated the PI-RADS to version 2.1, which is a standardized scoring system for performing, interpreting, and reporting the PCa with multiparametric MRI (mpMRI) (7). According to PI-RADS, the full mpMRI examination consists of at least two orthogonal planes T2WI images, axial T1WI images, axial diffusion-weighted images (DWI) and their apparent diffusion coefficient (ADC) values along with axial dynamic contrast-enhanced (DCE) images. However, several preliminary studies demonstrated that DCE plays only a secondary role in the transition zone (TZ) because it is not reliable for the differentiation between prostate cancer and benign prostatic hyperplasia. Moreover, it is also ignored in the peripheral zone (PZ) (8). Therefore*,* bpMRI has been investigated and demonstrated equivalent diagnostic performance to mpMRI while using PI-RADS, in which DCE was omitted thereafter this could decrease image acquisition time and costs, while retaining sufficient accuracy (9). Nevertheless, some studies showed that bpMRI had higher specificity but lower sensitivity as compared to mpMRI (9). Therefore, the combination of bpMRI and other clinical variables and biomarkers should be considered to improve the overall diagnostic performance. Recently, several simplified or revised scorings based on bpMRI had been proposed; however, these scorings have not been compared directly up to date (8,10–12). In this study, we aimed to compare the diagnostic performance of 3 scorings that based on bpMRI with PI-RADS v2.1. Additionally, we constructed integrated nomograms combining scorings based on bpMRI and clinical variables using multivariable logistic regression to identify csPCa.

**MATERIALS AND METHODS**

***Patient Selection***

This retrospective study was approved by our institutional review board and the requirement for written informed consent was waived. We reviewed the medical records of our institutional database to identify patients who had the suspicion of PCa (with PSA levels >4 ng/mL). Those who underwent mpMRI and subsequent MRGB between February 2017 and October 2021 were considered potentially eligible. Initially, 401 consecutive patients were identified, of them 77 were excluded for reasons as follows: 1) with PCa diagnosis or treatment prior to MRI (*n*=29); 2) incomplete systematic biopsy (*n*=14); and 3) insufficient clinical information (*n*=34). Finally, 324 patients with suspected PCa were included in this study, of which 217 were assigned to the training group and 107 were assigned to the validation group. **Figure 1** presents the detailed patient selection process.

***MRI Acquisition and Interpretation***

The prostate MRI examination was performed with a 3.0 T MRI scanner (Philips Ingenia, The Netherlands), and a pelvic 32-channel phased array coil was used for all patients. According to recommendations of PI-RADS v2.1 guideline, the following imaging protocols were used: axial turbo spin echo (TSE) T2 weighted image (T2WI, repetition time [TR]=3000ms; echo time [TE]=100ms; slice thickness=3.0 mm, the scanning field of view (FOV)=220mm×220mm, matrix=276×240), sagittal TSE T2WI (TR=6000ms, TE=77ms, slice thickness=3.0 mm, FOV=220mm×220mm, matrix=104×125), and axial DWI (TR=6000ms, TE=77ms, layer thickness=3.0mm, FOV =260mm×260mm, matrix=104×125) and multiple ***b*** values (*b*=0, 100, 1000, 2000 s/mm^2^).

All examinations were independently reviewed by two board-certified radiologists (*H.B.B.*, with 3 years of experience and *J.Y.M.*, with 7 years of experience), who were blinded to clinical information and pathologic results. Three scorings (Scoring 1, Scoring 2, and Scoring 3) derive from the PI-RADS to assess each lesion, in which the DCE sequence was omitted. Regarding Scoring 1, the final score was determined as follows: for PZ lesions, the DWI sequence is the dominant scoring sequence, and TZ lesions remain unchanged; for Scoring 2, T2WI replaces the DCE sequence to determine the final score for PZ lesions; however, when DWI=3 and when T2WI ≥ 4 the final score is upgraded to 4, and TZ lesions are unchanged; with respect to Scoring 3, the total score is determined by combining the T2WI and the DWI regardless of PZ and TZ lesions, details regarding these 3 scorings are summarized in **Table 1**.

***Biopsy and Histopathology***

All patients underwent systematic TRUS-guided biopsy after at least 4 weeks of the MRI examination, in which "10+x" cores were obtained in a double sextant pattern, sampling the lateral and medial portions of the apex, mid, and base of each hemi-gland. After examination of mpMRI, patients with suspicious lesions underwent fusion biopsy by using an ESAOTE Mylab Twice color Doppler ultrasound device (with a 7.5-MHz transrectal end-fire probe), which equipped with a Real-time Virtual Sonography imaging fusion system.

All fusion biopsies were performed by a urologist with 7 years of experience in prostate biopsy. Specimens (biopsy or radical prostatectomy) were assigned Gleason score (GS) by an expert genitourinary pathologist with more than 15 years of experience who was blinded to the MRI findings. All GS were assigned in concordance with the 2014 International Society of Urological Pathology consensus guideline (13). Clinically significant PCa was defined as GS ≥ 7 and tumor diameter ≥ 5 mm. Prostate volume (PV) was calculated according to the ellipsoid volume formula (transverse width × transverse length × longitudinal height × 0.52), and PSAD was calculated by dividing the tPSA level by PV (tPSA/PV), tumor size was primarily calculated from T2WI.

***Statistical Analysis***

For each scoring, we calculated their sensitivity and specificity along with their 95% confidence intervals (CI). The best threshold for each scoring that balanced sensitivity and specificity was calculated using Youden’s index (14). For Scoring 1 and Scoring 2, ≥4 was defined as positive, while for Scoring 3 positive was defined as ≥8. Subsequently, we used the univariable and multivariable logistic regression analysis to identify independent predictors associated with PCa, which includes age, total PSA (tPSA), free PSA (fPSA), PV, PSAD, and scorings. After conducting univariable logistic regression analysis for all potential clinical variables and biomarkers, we performed multivariable logistic regression analysis to investigate significant clinical factors for csPCa. For each scoring we constructed corresponding model (Model 1, Model 2, and Model 3) combined with clinical variables, overall diagnostic performance of AUCs were calculated and compared with DeLong’s test, the best was defined as the one with the largest AUC (15). A nomogram for the best combination in the multiple logistic regression analyses was generated for the prediction of csPCa. All analysis was performed using R statistical software (version 3.6.1).

**Results**

***Patient Characteristics***

The patient characteristics are presented in ***Table 2***. In the training group, 68 of 217 patients (31.3%) were diagnosed with csPCa based on the results from both targeted and/or systemic biopsies, whereas in the validation group 36 of 107 patients (33.6%) were diagnosed with csPCa.

***Diagnostic Performance of 3 Scorings Based on bpMRI***

For the detection of csPCa, the sensitivity and specificity for Scoring 1 at cutoff ≥4 were 64.7% (95% CI 52.2%-75.9%) and 80.5% (95% CI 73.3%-86.6%); for Scoring 2 were 86.8% (95% CI 76.4%-93.8%) and 73.2% (95% CI 65.3%-80.1%) at cutoff≥4; and for Scoring 3 were 61.8% (95% CI 49.2%-73.3%) and 80.5% (95% CI 73.3%-86.6%) at cutoff ≥8, respectively. The results showed that Scoring 2 had significantly higher sensitivity compared with Scoring 1 (P<0.001) and Scoring 2 (P=0.03) but with lower specificity (P<0.01 for both). **Figure 2** shows an example of one lesion categorized by 3 scorings.

***Logistic Regression Analysis***

Our univariate logistic regression analysis revealed that age (OR=1.06, 95% CI 1.03-1.10), PSA (OR=1.05, 95% CI 1.03-1.07), PV (OR=0.98, 95% CI 0.97-0.99), and PSAD (OR=34.27, 95% CI 9.87-119.02), and 3 scorings (OR=2.87, 95% CI 2.13-3.87 for Scoring 1; OR=3.51, 95% CI 2.49-4.95 for the Scoring 2; and OR=1.76, 95% CI 1.49-2.08 for Scoring 3) were significant predictors for csPCa. Nevertheless, as PSA and PV were strongly correlated with PSAD, which were excluded from the results. Finally, age, PSAD, and scorings were included in the multivariable logistic regression analyses. ***Table 3*** shows the details of univariate and multivariate logistic regression analyses. For 3 models, the AUCs were 0.88 (95% CI 0.83-0.93) for Model 1, 0.90 (95% CI 0.85-0.94) for Model 2, and 0.88 (95% CI 0.83-0.93) for Model 3 (**Figure 3**). For all models, the AUCs were significantly higher than using the scorings alone. We performed comparisons between these 3 models based on bpMRI, and the results suggested that Model 2 had significantly higher performance that Model 1 (P=0.04) and Model 3 (P=0.02) with DeLong’s test.

***Internal Validation***

On the validation data set, the AUCs were 0.88 (95% CI 0.81-0.95) for Model 1, 0.90 (95% CI 0.84-0.96) for Model 2, and 0.88 (95% CI 0.82-0.95) for Model 3. Delong’s test showed that the prediction of Model 2 was significantly higher as compared with Model 1 (P=0.04), but did not substantially different than Model 3 (P=0.05). A nomogram based on the combination of Scoring 2, age, and PSAD was constructed, in which points from each variable are added and a straight line from the total point score shows the probability of harboring csPCa (**Figure 4**).

**Discussion**

In this study, we evaluated 3 scoring systems based on bpMRI for the detection of csPCa. Our findings demonstrated that all three scorings exhibited high diagnostic performance, and the sensitivity and specificity for Scoring 1 were 64.7% and 80.5%, for Scoring 2 were 86.8% and 73.2%, and for Scoring 3 were 61.8% and 80.5%, respectively. Notably, with Scoring 1 significantly more csPCa were identified as compared with Scoring 2 (P<0.001) and Scoring 3 (P=0.03); however, the specificity was decreased substantially (P<0.001). In light of limited performance that merely depends on scoring systems, we constructed integrated nomograms combining clinical variables of age, PSAD, and scorings to identify csPCa lesions.

Our analysis revealed that all 3 models were substantially outperformed using scoring systems alone; moreover, we noted that Model 2 (AUC 0.90) was significantly superior to Model 1 (AUC 0.88, P=0.04) and Model 2 (AUC 0.88, P=0.02). To validate these three nomograms, we analyzed 107 patients (36 pathologically proved csPCa) in the validation group. The results showed that the diagnostic performance was consistent with results from the training group, and the AUC for the three models were 0.88, 0.90, and 0.88, respectively. Furthermore, Model 2 was significantly superior to Model 1 while compared using the AUC (P=0.04); however, the difference was not statistically significant as compared with Model 3 (P=0.05). Previous studies had reported that combining PI-RADS and other clinical variables or biomarkers was beneficial for improving the diagnostic performance for the detection of PCa; however, most of the nomograms or models from these studies were based on mpMRI and have not been validated in another cohort set (11,16,17). In this study, we independently validated our nomograms using a different dataset, and the results showed the models generalized well.

Given the inconvenience and limitation of mpMRI, several preliminary studies have investigated bpMRI that omit the DCE sequence, as which is not need intravenous contrast media and is faster (up to 15 min) (18). Many studies reported that the bpMRI has comparable performance with mpMRI, and the DCE was not necessary or secondary, especially for PZ lesions; however, the role of DCE imaging in the prediction of PCa has been the topic of much discussion. The use of DCE imaging is increasingly controversial, and more and more studies suggested that it could be abandoned (8,10,19,20). A previous meta-analysis that included 11 head-to-head studies comparing bpMRI and mpMRI showed that the former had lower sensitivity but higher specificity (9). Nevertheless, Greer et al. demonstrated that DCE could improve the probability of cancer detection of PI-RADS category 2, 3, and 4 for PZ lesions; however, this study reported that the high rate of DEC positivity may lead to higher false-positive findings (10). In the current study, our results showed that without DCE, bpMRI still yielded high diagnostic performance. Furthermore, fewer MRI sequences led to the higher inter-reader agreement, and a simplified scoring system could benefit those radiologists with less experience.

One problem that deserves attention is that no widely accepted standard for how bpMRI addresses the omission of DCE. Some studies proposed that replace the DCE sequence with T2WI (21–23), while others recommend abandoning the DCE sequence (11). In our study, three different modified bpMRI scorings were evaluated and have been compared with each other, and our results were consistent with the study of Boesen et al. (24). In that study, scoring of lesions in the PZ only depended on DWI findings (dominant sequence), and an equivocal score of 3 was not potentially upgraded to a score of 4 for lacking positive DCE findings. In the study of De Visschere et al., the absence of DCE was resolved by using T2WI for PZ lesions while the DWI score was 3, and three different threshold scores were used for T2-positivity (≥3, ≥4, and 5).

Our study has some limitations. First, this was a single-center retrospective study and patient selection bias may limit the generalizability of our study, thus the results and conclusions need validation in prospective multi-center studies with a larger number of patients. Second, we did not report PZ and TZ lesions separately. Considering different dominant MRI sequences for these 2 anatomy zones, the detailed diagnostic for PZ and TZ lesions should be investigated in future study. Third, use of systematic and/or MRI-TRUS fusion targeted biopsy as the reference standard may lead to some lesions with positive pathology missed as which showed negative in MRI. Last, in this study we did not report the inter-reader agreement between radiologists, which is important for the standardized scoring system as it relates to reducing the variability of interpretation. However, because our main goal was to develop and validate nomogram for csPCa, investigation of the inter-reader agreement was beyond the scope of our study.

**Conclusions**

In this study, we compared three scorings that based on bpMRI and the results showed that Scoring 2 performed better than the remaining two scorings. Additionally, 3 models were constructed using 3 scorings with age and PASD and Model 2 that based on Scoring 2 yielded the highest performance.

**Declarations**

**Ethics approval and consent to participate**

Not applicable.

**Consent for publication**

Patients data were anonymized. Not applicable.

**Availability of data and materials**

The data that support the findings of this study are available from the corresponding author upon reasonable request.

**Competing interests**

Authors declare no conflict of interest.

**Funding**

Not applicable.

**Author contributions** *H.B.B.*: Manuscript writing, Data collection, Statistical analysis. *Z.Y.Y.*: Manuscript writing, Analysis and interpretation of data. *Z.H.L.*: Data collection. *J.Y.M.*: Project development, Supervision.

**Acknowledgments**

Not applicable.

**Reference**

1. GBD 2017 Disease and Injury Incidence and Prevalence Collaborators. Global, regional, and national incidence, prevalence, and years lived with disability for 354 diseases and injuries for 195 countries and territories, 1990-2017: a systematic analysis for the Global Burden of Disease Study 2017. *Lancet Lond Engl* (2018) 392:1789–1858. doi: 10.1016/S0140-6736(18)32279-7

2. Foreman KJ, Marquez N, Dolgert A, Fukutaki K, Fullman N, McGaughey M, Pletcher MA, Smith AE, Tang K, Yuan C-W, et al. Forecasting life expectancy, years of life lost, and all-cause and cause-specific mortality for 250 causes of death: reference and alternative scenarios for 2016-40 for 195 countries and territories. *Lancet Lond Engl* (2018) 392:2052–2090. doi: 10.1016/S0140-6736(18)31694-5

3. Tamada T, Sone T, Higashi H, Jo Y, Yamamoto A, Kanki A, Ito K. Prostate Cancer Detection in Patients With Total Serum Prostate-Specific Antigen Levels of 4-10 ng/mL: Diagnostic Efficacy of Diffusion-Weighted Imaging, Dynamic Contrast-Enhanced MRI, and T2-Weighted Imaging. *AJR Am J Roentgenol* (2011) 197:664–70. doi: 10.2214/AJR.10.5923

4. Vilanova JC, Barceló-Vidal C, Comet J, Boada M, Barceló J, Ferrer J, Albanell J. Usefulness of Prebiopsy Multifunctional and Morphologic MRI Combined With Free-to-Total Prostate-Specific Antigen Ratio in the Detection of Prostate Cancer. *Am J Roentgenol* (2011) 196:W715–W722. doi: 10.2214/AJR.10.5700

5. Delongchamps NB, Rouanne M, Flam T, Beuvon F, Liberatore M, Zerbib M, Cornud F. Multiparametric magnetic resonance imaging for the detection and localization of prostate cancer: combination of T2‐weighted, dynamic contrast‐enhanced and diffusion‐weighted imaging. *Bju Int* (2011) 107:1411–1418. doi: 10.1111/j.1464-410X.2010.09808.x

6. Ahmed HU, Bosaily AE-S, Brown LC, Gabe R, Kaplan R, Parmar MK, Collaco-Moraes Y, Ward K, Hindley RG, Freeman A, et al. Diagnostic accuracy of multi-parametric MRI and TRUS biopsy in prostate cancer (PROMIS): a paired validating confirmatory study. *The Lancet* (2017) 389:815–822. doi: 10.1016/S0140-6736(16)32401-1

7. Turkbey B, Rosenkrantz AB, Haider MA, Padhani AR, Villeirs G, Macura KJ, Tempany CM, Choyke PL, Cornud F, Margolis DJ, et al. Prostate Imaging Reporting and Data System Version 2.1: 2019 Update of Prostate Imaging Reporting and Data System Version 2. *Eur Urol* (2019) 76:340–351. doi: 10.1016/j.eururo.2019.02.033

8. De Visschere P, Lumen N, Ost P, Decaestecker K, Pattyn E, Villeirs G. Dynamic contrast-enhanced imaging has limited added value over T2-weighted imaging and diffusion-weighted imaging when using PI-RADSv2 for diagnosis of clinically significant prostate cancer in patients with elevated PSA. *Clin Radiol* (2017) 72:23–32. doi: 10.1016/j.crad.2016.09.011

9. Niu X, Chen X, Chen Z, Chen L, Li J, Peng T. Diagnostic Performance of Biparametric MRI for Detection of Prostate Cancer: A Systematic Review and Meta-Analysis. *Am J Roentgenol* (2018) 211:369–378. doi: 10.2214/AJR.17.18946

10. Greer MD, Shih JH, Lay N, Barrett T, Kayat Bittencourt L, Borofsky S, Kabakus IM, Law YM, Marko J, Shebel H, et al. Validation of the Dominant Sequence Paradigm and Role of Dynamic Contrast-enhanced Imaging in PI-RADS Version 2. *Radiology* (2017) 285:859–869. doi: 10.1148/radiol.2017161316

11. Lee S, Oh YT, Jung DC, Cho NH, Choi YD, Park SY. Combined Analysis of Biparametric MRI and Prostate-Specific Antigen Density: Role in the Prebiopsy Diagnosis of Gleason Score 7 or Greater Prostate Cancer. *Am J Roentgenol* (2018) 211:W166–W172. doi: 10.2214/AJR.17.19253

12. Rosenkrantz AB, Babb JS, Taneja SS, Ream JM. Proposed Adjustments to PI-RADS Version 2 Decision Rules: Impact on Prostate Cancer Detection. *Radiology* (2017) 283:119–129. doi: 10.1148/radiol.2016161124

13. Epstein JI, Amin MB, Reuter VE, Humphrey PA. Contemporary Gleason Grading of Prostatic Carcinoma: An Update With Discussion on Practical Issues to Implement the 2014 International Society of Urological Pathology (ISUP) Consensus Conference on Gleason Grading of Prostatic Carcinoma. *Am J Surg Pathol* (2017) 41:e1–e7. doi: 10.1097/PAS.0000000000000820

14. Hughes G. Youden’s index and the weight of evidence. *Methods Inf Med* (2015) 54:198–199. doi: 10.3414/ME14-04-0003

15. DeLong ER, DeLong DM, Clarke-Pearson DL. Comparing the areas under two or more correlated receiver operating characteristic curves: a nonparametric approach. *Biometrics* (1988) 44:837–845.

16. Distler FA, Radtke JP, Bonekamp D, Kesch C, Schlemmer H-P, Wieczorek K, Kirchner M, Pahernik S, Hohenfellner M, Hadaschik BA. The Value of PSA Density in Combination with PI-RADS^TM^ for the Accuracy of Prostate Cancer Prediction. *J Urol* (2017) 198:575–582. doi: 10.1016/j.juro.2017.03.130

17. Washino S, Okochi T, Saito K, Konishi T, Hirai M, Kobayashi Y, Miyagawa T. Combination of prostate imaging reporting and data system (PI-RADS) score and prostate-specific antigen (PSA) density predicts biopsy outcome in prostate biopsy naïve patients. *BJU Int* (2017) 119:225–233. doi: 10.1111/bju.13465

18. Sherrer RL, Glaser ZA, Gordetsky JB, Nix JW, Porter KK, Rais-Bahrami S. Comparison of biparametric MRI to full multiparametric MRI for detection of clinically significant prostate cancer. *Prostate Cancer Prostatic Dis* (2019) 22:331–336. doi: 10.1038/s41391-018-0107-0

19. Iwazawa J, Mitani T, Sassa S, Ohue S. Prostate cancer detection with MRI: is dynamic contrast-enhanced imaging necessary in addition to diffusion-weighted imaging? *Diagn Interv Radiol* (2011) 17:243–8.

20. Rud E, Baco E. Re: Jeffrey C. Weinreb, Jelle O. Barentsz, Peter L. Choyke, et al. PI-RADS Prostate Imaging - Reporting and Data System: 2015, Version 2. Eur Urol 2016;69:16-40: Is Contrast-enhanced Magnetic Resonance Imaging Really Necessary When Searching for Prostate Cancer? *Eur Urol* (2016) 70:e136. doi: 10.1016/j.eururo.2016.04.017

21. Pan J, Su R, Cao J, Zhao Z, Ren D, Ye S, Huang R, Tao Z, Yu C, Jiang J, et al. Modified Predictive Model and Nomogram by Incorporating Prebiopsy Biparametric Magnetic Resonance Imaging With Clinical Indicators for Prostate Biopsy Decision Making. *Front Oncol* (2021) 11:740868. doi: 10.3389/fonc.2021.740868

22. Kuhl CK, Bruhn R, Krämer N, Nebelung S, Heidenreich A, Schrading S. Abbreviated Biparametric Prostate MR Imaging in Men with Elevated Prostate-specific Antigen. *Radiology* (2017) 285:493–505. doi: 10.1148/radiol.2017170129

23. Scialpi M, Aisa MC, D’Andrea A, Martorana E. Simplified Prostate Imaging Reporting and Data System for Biparametric Prostate MRI: A Proposal. *AJR Am J Roentgenol* (2018) 211:379–382. doi: 10.2214/AJR.17.19014

24. Boesen L, Thomsen FB, Nørgaard N, Løgager V, Balslev I, Bisbjerg R, Thomsen HS, Jakobsen H. A predictive model based on biparametric magnetic resonance imaging and clinical parameters for improved risk assessment and selection of biopsy-naïve men for prostate biopsies. *Prostate Cancer Prostatic Dis* (2019) doi: 10.1038/s41391-019-0149-y

*Table 1. Three scorings based on bpMRI*

*Table 2. Patient characteristics.*

*Table 3. Univariate and multivariate logistic regression analyses.*

*Figure 1. Flowchart of the study population with the exclusion criteria. csPCa, clinically significant prostate cancer.*

Figure 2. *A 63 years old man with elevated PSA of 11.1ng/ml and PSAD of 0.27ng/ml/ml. (a)*

*A marked focal lesion (white arrow) of 11 mm in the right of the PZ with low signal intensity on T2WI; (b) high signal intensity on high b-value image of the DWI; (c) on the ADC map, which showed hyperintense; (d) histopathologic image, Gleason score of 4 + 4 at prostatectomy. This lesion was assigned as score 3, 4, and 7 according to Scoring 1, Scoring 2, and Scoring 3, respectively.*

*Figure 3. ROC for the comparison 3 models. A, area under the ROC curve for 3 scorings; B, area under the ROC curve for 3 models. AUC, area under the ROC curve.*

*Figure 4. Construction of a nomogram for predicting the probability of clinically significant prostate cancer. bpMRI, biparametric magnetic resonance imaging; PSAD, prostate-specific antigen density.*
