# Supplementary material for: A nomogram based on biparametric magnetic resonance imaging for detection of clinically significant prostate cancer in biopsy-naïve patients
Source: Cancer Imaging. 2023 Sep 4;23:82. doi: 10.1186/s40644-023-00606-2 (PMC10478308; doi:10.1186/s40644-023-00606-2)
Supplement: Supplementary file 2 — Supplementary Material 2 [file 40644_2023_606_MOESM2_ESM.docx]

**Table 2 Diagnostic Performance of Scorings and Models for Training Cohort**

| **Indicator** | **Cutoff** | Sensitivity (95% CI) | Specificity (95% CI) | | AUC (95% CI) | | ***P*** |
| --- | --- | --- | --- | --- | --- | --- | --- |
| Scoring 1 | ≥4 | 64.7% (52.2%-75.9%) | | 80.5% (73.3%-86.6%) | | 0.81 (0.76-0.87) | 0.03 |
| Scoring 2 | ≥4 | 86.8% (76.4%-93.8%) | | 73.2% (65.3%-80.1%) | | 0.83 (0.78-0.89) | REF |
| Scoring 3 | ≥8 | 61.8% (49.2%-73.3%) | | 80.5% (73.3%-86.6%) | | 0.81 (0.75-0.87) | <0.01 |

**Abbreviations**: AUC, area under the receiver operating characteristic curve; CI, confidence interval; REF, reference.
