# Supplementary material for: A nomogram based on biparametric magnetic resonance imaging for detection of clinically significant prostate cancer in biopsy-naïve patients
Source: Cancer Imaging. 2023 Sep 4;23:82. doi: 10.1186/s40644-023-00606-2 (PMC10478308; doi:10.1186/s40644-023-00606-2)
Supplement: Supplementary file 3 — Supplementary Material 3 [file 40644_2023_606_MOESM3_ESM.docx]

**Reviewer #1:**

Authors have retrospectively reviewed mpMRI of 324 patients and disregarded the DCE sequences to obtain the diagnostic performance of bpMRI in the detection csPCA. Additionally they have created three clinical decision models based on nomograms in the detection of csPCA and found good performance of one such models. They concluded that bpMRI without DCE may be accurate in diagnosis of csPCA especially if combined with other clinical parameters.

Abstract:

"We assessed the diagnostic performance of 3 scorings…" should be "We assessed the diagnostic performance of three bp-MRI based scorings…"

***Response***: Thank you for your constructive advice. We have revised this sentence as advised.

"Furthermore, and 3 models…" should be " Furthermore, 3 models…"

***Response***: We deleted the redundant “and”.

The abstract should mention the diagnostic performance of the bpMRI based scoring systems before it mentions the diagnostic performance of the models.

***Response***: We have revised it and added following contents before the diagnostic performance of the models:

“The sensitivity and specificity for Scoring 1 were 64.7% (95% CI 52.2%-75.9%) and 80.5% (95% CI 73.3%-86.6%); for Scoring 2 were 86.8% (95% CI 76.4%-93.8%) and 73.2% (95% CI 65.3%-80.1%); and for Scoring 3 were 61.8% (95% CI 49.2%-73.3%) and 80.5% (95% CI 73.3%-86.6%), respectively.”

The conclusion says "scoring 2" has the best performance. It should be model 2.

***Response***: We found that the description was vague, and this sentence was revised as follows:

“All three scorings had favorite diagnostic accuracy. While in conjunction with age and PSAD the prediction power was significantly improved, and the Model 2 that based on Scoring 2 yielded the highest performance.”

**Introduction:**

"However, time-consuming…application of the PIRADS" This is partly true. What the authors are referring to specifically the DCE images. Please modify accordingly.

***Response***: We revised this section to:

“However, preliminary studies demonstrated that DCE plays only a secondary role in transition zone (TZ) because it is not reliable for the differentiation between prostate cancer and benign prostatic hyperplasia. Moreover, it is also ignored in the peripheral zone (PZ). Therefore, bpMRI has been investigated and demonstrated equivalent diagnostic performance to mpMRI while using PI-RADS v2, in which DCE was omitted thereafter this could decrease image acquisition time and costs, while retaining sufficient accuracy.”

Tamada et al actually showed that the diagnostic sensitivity was higher for mpMRI but diagnostic specificity was significantly higher for bpMRI. Please correct accordingly. Also this makes the premise of this study a bit superfluous since adding further tests to a highly specific test is unlikely to increase specificity. Please discuss this.

***Response***: We revised this sentence and cited a meta-analysis instead of a single article:

“Nevertheless, some studies showed that bpMRI had higher specificity but lower sensitivity as compared to mpMRI.”

Moreover, we have spent a paragraph to discuss bpMRI vs. mpMRI.

**Materials and Methods:**

In the "MRI Acquisition and Interpretation" section please use 'slice thickness' instead of 'layer thickness'.

***Response***: We have revised accordingly.

For the purpose of calculation of sensitivity or specificity, definition of 'test positive' an 'test negative' needs to be determined (such PIRADS 3 or higher as test positive). Please mention this.

***Response***: We have added the following sentence to define positive: “For Scoring 1 and Scoring 2, ≥4 was defined as positive, while for Scoring 3 positive was defined as ≥8.”

For ordinal variables such as mpMRI score, a ROC curve is inappropriate. Please delete this. Simply mentioning sensitivity, specificity and Youden index should be enough.

***Response***: We have revised it as advised:

“For each scoring, we calculated their sensitivity and specificity along with their 95% CI. The best threshold for each scoring that balanced sensitivity and specificity was calculated using Youden’s index.”

In addition to Method section, we have revised all relevant description in Results, Abstract, and Discussion.

And the ROC analysis was used only to models:

“For each scoring we constructed corresponding model (Model 1, Model 2, and Model 3) combining with clinical variables, overall diagnostic performance of AUCs were calculated and compared with DeLong’s test, the best was defined as the one with the largest AUC.”

**Results:**

"...in the validation group 34 patients (46.6%) were diagnosed with csPCa." Table 1 says the number of csPCA patients is 36 (33.6%). Please correct this.

***Response***: It should be “36 (33.6%)”, we have corrected it.

"The mean age was significant difference…" should be " The mean age was significantly different…"

***Response***: We have revised this sentence.

The values that are already mentioned in Table 1 need not be repeated in text.

***Response***: We have revised and only kept some important values:

“In the training group, 68 of 217 patients (31.3%) were diagnosed with csPCa based on the results from both targeted and/or systemic biopsies, whereas in the validation group 36 of 107 patients (33.6%) were diagnosed with csPCa. The patient characteristics are presented in ***Table 1***.”

**Discussion:**

Seems appropriate.

**Figures:**

Figure 2 showing the ROC curve for mpMRI score is inappropriate as mentioned earlier since it is an ordinal variable. Observation of the curve shows that there are large slanted segments suggesting the calculation is not properly made. Compare this with the second curve showing performance of nomogram scores which has no oblique segments suggesting that it is an appropriate calculation. Furthermore, the x-axis of the ROC curve should have the false positive rate (1-specificity) and not specificity. Please delete the first diagram and correct the annotation in the second.

***Response***: We have removed the Figure 2, and revised the X-axis to “1-Specificity”.

**Charts:**

Table 2 is redundant. Please delete it. Instead I would like to see the bpMRI scoring system included in a table in the main article.

***Response***: We Removed the Table 2, then added a Table 1 for bpMRI scoring system.

References: Seems okay.

**Reviewer #2:**

Well written manuscript.

Grammatical revisions recommended.

***Response***: Thank you for your constructive advice.

We have scrutinized the whole article and made significant revision.

It's unclear which parameters were employed for each of the scoring systems. Kindly provide the parameters used for each of the 3 scoring systems in tabulated form.

***Response***: We added a table (Table 1) to provide the details on scoring.

Representative images would be appreciated.

***Response***: We added a figure (Figure 2) with representative images.

Thank you.
